# Information Length Analysis of Linear Autonomous Stochastic Processes

**DOI:** 10.3390/e22111265

**Published:** 2020-11-07

**Authors:** Adrian-Josue Guel-Cortez, Eun-jin Kim

**Affiliations:** Centre for Fluid and Complex Systems, Coventry University, Priory St, Coventry CV1 5FB, UK; ad3116@coventry.ac.uk

**Keywords:** non-equilibrium, stochastic processes, time-dependent PDF, information length, information geometry, entropy, fluctuations

## Abstract

When studying the behaviour of complex dynamical systems, a statistical formulation can provide useful insights. In particular, information geometry is a promising tool for this purpose. In this paper, we investigate the information length for *n*-dimensional linear autonomous stochastic processes, providing a basic theoretical framework that can be applied to a large set of problems in engineering and physics. A specific application is made to a harmonically bound particle system with the natural oscillation frequency ω, subject to a damping γ and a Gaussian white-noise. We explore how the information length depends on ω and γ, elucidating the role of critical damping γ=2ω in information geometry. Furthermore, in the long time limit, we show that the information length reflects the linear geometry associated with the Gaussian statistics in a linear stochastic process.

## 1. Introduction

Stochastic processes are common in nature or laboratories, and play a major role across traditional disciplinary boundaries (e.g., see [1,2]). These stochastic processes often exhibit complex temporal behaviour and even the emergence of order (self-organization). The latter can also be artificially designed to complete an orderly task (guided self-organization) [3,4,5,6]. In order to study and compare the dynamics of different stochastic processes and self-organization, it is valuable to utilize a measurement which is independent of any specifics of a system [7,8,9,10,11] (e.g., physical variables, units, dimensions, etc.). This can be achieved by using information theory based on probability density functions (PDFs) and working in terms of information content or information change, e.g., by quantifying the statistical difference between two states [12,13,14]. Mathematically, we do this by assigning a metric to probability and by using the notion of ‘length’ or ‘distance’ in the statistical space.

One method of measuring the information content in a system is utilizing the Fisher information, which represents the degree of certainty, or order. The opposite is entropy, which is a popular concept for the uncertainty or amount of disorder. Comparing entropy at different times then gives a measure of the difference in information content between the two states, which is known as relative entropy (e.g., see [15]). Another example is the Wasserstein metric [16,17], which provides an exact solution to the Fokker-Planck equation for a gradient flow subject to the minimization of the energy functional defined as the sum of the entropy and potential energy [18,19,20]. This metric has units of a physical length in comparison with other metrics, for instance the dimensionless statistical distance based on the Fisher information metric [21,22,23]. Interestingly, there is a link between the Fisher information and the Wasserstein distance [24]. Furthermore, the relative entropy can be expressed by the integral of the Fisher information along the same path [25].

Although quite useful, the relative entropy lacks the locality of a metric as it concerns only about the difference between given two PDFs. For instance, when these two PDFs represent the two states at different times, the relative entropy between them tells us nothing about how one PDF evolves to the other PDF over time or what intermediate states a system passes through between the two PDFs. As a result, it can only inform us of the changes that affect the overall system evolution [26]. To overcome this limitation, the information length L(t) was proposed in recent works, which quantifies the total number of different states that the system evolves through in time [27,28]. This means that the information length is a measure that depends on the evolution path between two states (PDFs). Its formulation allows us to measure local changes in the evolution of the system as well as providing an intriguing link between stochastic processes and geometry [26].

For instance, the relation between the information length $L_{\infty }=L\left(t\to \infty \right)$ and the mean value of the initial PDF for the fixed values of all other parameters was invoked as a new way of mapping out an attractor structure in a relaxation problem where any initial PDF relaxes into its equilibrium PDF in the long time limit. Specifically, for the Ornstein-Uhlenbeck (O-U) process driven by a Gaussian white-noise (which is a linearly damped, relaxation problem), $L_{\infty }$ increases linearly with the distance between the mean position of an initial PDF and the stable equilibrium point for further details, see [28,29], with its minimum value zero at the stable equilibrium point. This linear dependence manifests that the information length preserves the linear geometry of the underlying Gaussian process, which is lost in other metrics [26]. For a nonlinear stochastic process with nonlinear damping, $L_{\infty }$ still takes its minimum value at the stable equilibrium point but exhibits a power-law dependence on the distance between the mean value of an initial PDF and the stable equilibrium point. In contrast, for a chaotic attractor, $L_{\infty }$ changes abruptly under an infinitesimal change of the mean value of an initial PDF, reminiscent of the sensitive dependence on initial conditions of the Lyapunov exponent [30]. These results suggest that $L_{\infty }$ elucidates how different (non)linear forces affect (information) geometry.

With the above background in mind, this paper aims to extend the analysis of the information length of the O-U process to an arbitrary *n*-th order linear autonomous stochastic processes, providing a basic theoretical framework to be utilized in a large set of problems in both engineering and physics. In particular, we provide a useful analytical result that defines the information diagnostics as a function of the covariance matrix and the mean vector of the system, which enormously reduces the computational cost of numerical simulations of high-order systems.

This is followed by a specific application to a harmonically bound particle system (Kramers equation) for the position *x* and velocity $v=\frac{dx}{dt}$, with the natural oscillation frequency ω, subject to a damping constant γ and a Gaussian white-noise (short-correlated). We find an exact time-dependent joint PDF p(x,v,t) starting from an initial Gaussian PDF which has a finite-width. Note that as far as we are aware of, our result p(x,v,t) is original since in literature, the calculation was done only for the case of a delta-function initial PDF. Since this process is governed by the two variables, *x* and *v*, we investigate how $L_{\infty }$ depends on their initial mean values $\langle x_{0}\rangle$ and $\langle v_{0}\rangle$. Here, the angular brackets denote the average. Furthermore, the two characteristic time scales associated with ω and γ raise the interesting question as to their role in $L_{\infty }$. Thus, we explore how the information length depends on ω and γ. Our principle results are as follows: (i) $L_{\infty }$ tends to increase linearly with either the deviation of initial mean position $\langle x_{0}\rangle$ or the initial mean velocity $\langle v_{0}\rangle$ from their equilibrium values 〈x(0)〉=〈v(0)〉=0; (ii) a linear geometry is thus preserved for our linearly coupled stochastic processes driven by a Gaussian noise; (iii) $L_{\infty }$ tends to take its minimum value near the critical damping γ=2ω for the same initial conditions and other parameters.

The remainder of this paper is organized as follows: Section 2 presents the basic concept of information length and the formulation of our problem. In Section 3, our main theoretical results are provided (see also Appendix A). In Section 4, we apply the results in Section 3 to analyze a harmonically bound particle system with the natural oscillation frequency ω subject to a damping constant γ and a Gaussian white-noise. Finally, Section 5 contains our concluding remarks.

To help readers, we here provide a summary of our notations: R and C are the sets of real and complex numbers, respectively. $x\in R^{n}$ represents a column vector x of real numbers of dimension *n*, $A\in R^{n\times n}$ represents a real matrix of dimension n×n, tr(A) corresponds to the trace of the matrix A, $A^{T}$ and $A^{-1}$ are the transpose and inverse of matrix A, respectively. (Bold-face letters are used to represent vectors and matrices.) In some places, $\partial_{t}$ or the prime both are used for the partial derivative with respect to time. Besides, $i=\sqrt{-1}$ and for s∈C, $L^{-1}\left[F(s)\right]=\frac{1}{2\pi i}\lim_{b\to \infty }\int_{a-bi}^{a+bi}e^{st}F\left(s\right)ds$ corresponds to the inverse Laplace transform of the complex function F(s). Finally, the average of a random vector x is denoted by 〈x〉.

## 2. Preliminaries

### 2.1. Information Length

As noted in Section 1, the information length [26,27,31] is a dimensionless measurement of the total number of statistically different states that a system passes through in time in non-equilibrium processes. We cannot overemphasize that it is a measure that depends on the evolution of the system, being a useful index for understanding the information geometry underlying non-equilibrium processes. For example, for a time-dependent PDF p(x,t) of one stochastic variable *x*, the information length L(t) is the total information change between time 0 and *t*, and is defined by

(1) $$L\left(t\right)=\int_{0}^{t}\frac{dt_{1}}{\tau (t_{1})}=\int_{0}^{t}dt_{1}\sqrt{E(t_{1})}=\int_{0}^{t}dt_{1}\sqrt{\int_{-\infty }^{\infty }dx\frac{1}{p(x,t_{1})}{\left[\frac{\partial p(x,t_{1})}{\partial t_{1}}\right]}^{2}}.$$

Here, $E(t_{1})=\int_{-\infty }^{\infty }dx\frac{1}{p(x,t_{1})}{\left[\frac{\partial p(x,t_{1})}{\partial t_{1}}\right]}^{2}$ is the square of the information velocity (recalling we are working with the unit where the distance given by the information length has no dimension). As we can see, to define the information length, we compute the dynamic time unit $\tau \left(t\right)=\frac{1}{\sqrt{E}}$, which quantifies the correlation time over which the PDF p(x,t) changes. Besides, τ serves as the time unit in the statistical space. Alternatively, the information velocity $\frac{1}{\tau (t_{1})}$ quantifies the (average) rate of change of information in time.

### 2.2. Problem Formulation

We consider the following linear autonomous process

(2) $$\dot{x}\left(t\right)=Ax\left(t\right)+\Gamma \left(t\right).$$

Here, A is an n×n constant real matrix; $\Gamma \in R^{n}$ is a stochastic driving given by a *n* dimensional vector of δ-correlated Gaussian noises $\Gamma_{i}$ (i=1,2,...n), with the following statistical property

(3) $$\langle \Gamma_{i}\left(t\right)\rangle =0,\langle \Gamma_{i}\left(t\right)\Gamma_{j}\left(t_{1}\right)\rangle =2D_{ij}\delta \left(t-t_{1}\right),D_{ij}=D_{ji},\forall i,j=1,\cdots ,n.$$

Note that $D_{ii}$ represents the strength of the *i*-th stochastic noise while $D_{ij}$ for i≠j denotes the correlation between *i*-th and *j*-th noises (i.e., random fluctuations). Then, from the joint PDF p(x,t), we define the information length L of system (2) by the following integral

(4) $$L\left(t\right)=\int_{0}^{t}dt_{1}\sqrt{\int_{-\infty }^{\infty }dx\frac{1}{p(x,t_{1})}{\left[\frac{\partial p(x,t_{1})}{\partial t_{1}}\right]}^{2}}=\int_{0}^{t}dt_{1}\sqrt{E},$$

where $E=\int_{-\infty }^{\infty }dx\frac{1}{p(x,t_{1})}{\left[\frac{\partial p(x,t_{1})}{\partial t_{1}}\right]}^{2}$ is the square of the information velocity.

The first goal of this paper is to provide theoretical results for the information length (4) for the system (2) and (3). This is done in the following Section 3.

## 3. General Analytical Results

In the section, we provide the analytical results for Problem 2.1, summarizing the main steps required to calculate information length (4). To this end, we assume that an initial PDF is Gaussian and then take the advantage of the fact that a linear process driven by a Gaussian noise with an initial Gaussian PDF is always Gaussian. The joint PDF for (2) and (3) is thus Gaussian, whose form is provided below.

**Proposition** **1** (Joint probability). *The system (2) and (3) for a Gaussian random variable x at any time t has the following joint PDF*

(5) $$p\left(x,t\right)=\frac{1}{\sqrt{\det(2\pi \Sigma )}}e^{-\frac{1}{2}{\left(x-\langle x(t)\rangle \right)}^{T}\Sigma^{-1}\left(x-\langle x(t)\rangle \right)},$$

*where*

(6) $$\begin{array}{ccc} \langle x(t)\rangle & = & e^{At}\langle x\left(0\right)\rangle , \end{array}$$

(7) $$\begin{array}{ccc} \Sigma (t) & = & e^{At}\langle \delta x\left(0\right)\delta x{\left(0\right)}^{T}\rangle e^{A^{T}t}+2\int_{0}^{t}e^{A(t-t_{1})}De^{A^{T}\left(t-t_{1}\right)}dt_{1}, \end{array}$$

*and $D\in R^{n\times n}$ is the matrix of elements $D_{ij}$. Here, 〈x(t)〉 is the mean value of x(t) while *Σ* is the covariance matrix.*

**Proof.**  For a Gaussian PDF of x, all we need to calculate are the mean and covariance of x and substitute them in the general expression for multi-variable Gaussian distribution (5). To this end, we first write down the solution of Equation (2) as follows

(8) $$x\left(t\right)=e^{At}x\left(0\right)+\int_{0}^{t}e^{A(t-t_{1})}\Gamma \left(t_{1}\right)dt_{1}.$$

By taking the average of Equation (8), we find the mean value of x(t) of (8) as follows

(9) $$\langle x\left(t\right)\rangle =\langle e^{At}x\left(0\right)\rangle +\int_{0}^{t}e^{A(t-t_{1})}\langle \Gamma \left(t_{1}\right)\rangle dt_{1}=e^{At}\langle x\left(0\right)\rangle ,$$

which is Equation (6). On the other hand, to find covariance Σ(t), we let x=〈x〉+δx, and use the property 〈δx(0)Γ(t)〉=0 to find

(10) $$\begin{array}{ccc} \Sigma (t) & = & \langle \delta x\delta x^{T}\rangle \\ & = & \langle \left(e^{At}\delta x\left(0\right)+\int_{0}^{t}e^{A(t-t_{2})}\langle \Gamma \left(t_{2}\right)\rangle dt_{2}\right){\left(e^{At}\delta x\left(0\right)+\int_{0}^{t}e^{A(t-t_{1})}\langle \Gamma \left(t_{1}\right)\rangle dt_{1}\right)}^{T}\rangle \\ & = & \langle \left(e^{At}\delta x\left(0\right)+\int_{0}^{t}e^{A(t-t_{2})}\Gamma \left(t_{2}\right)dt_{2}\right)\left(\delta x{\left(0\right)}^{T}e^{A^{T}t}+\int_{0}^{t}\Gamma {\left(t_{1}\right)}^{T}{\left(e^{A(t-t_{1})}\right)}^{T}dt_{1}\right)\rangle \\ & = & e^{At}\langle \delta x\left(0\right)\delta x{\left(0\right)}^{T}\rangle e^{A^{T}t}+\langle \left(\int_{0}^{t}e^{A(t-t_{2})}\Gamma \left(t_{2}\right)dt_{2}\right)\left(\int_{0}^{t}\Gamma {\left(t_{1}\right)}^{T}e^{A^{T}\left(t-t_{1}\right)}dt_{1}\right)\rangle \\ & = & e^{At}\langle \delta x\left(0\right)\delta x{\left(0\right)}^{T}\rangle e^{A^{T}t}+\int_{0}^{t}\int_{0}^{t}e^{A(t-t_{2})}\langle \Gamma \left(t_{2}\right)\Gamma {\left(t_{1}\right)}^{T}\rangle e^{A^{T}\left(t-t_{1}\right)}dt_{2}dt_{1}\\ & = & e^{At}\langle \delta x\left(0\right)\delta x{\left(0\right)}^{T}\rangle e^{A^{T}t}+2\int_{0}^{t}e^{A(t-t_{1})}De^{A^{T}\left(t-t_{1}\right)}dt_{1}. \end{array}$$

Here δx(0)=δx(t=0) is the initial fluctuation at t=0. Equation (10) thus proves Equation (7). Substitution of Equations (6) and (7) in Equation (5) thus gives us a joint PDF p(x,t) ☐

Next, in order to calculate the information length from the joint PDF p(x,t) in Equation (5), we now use the following Theorem:

**Theorem** **1** (Information Length). *The information length of the joint PDF of system (2) and (3) is given by the following integral*

(11) $$L\left(t\right)=\int_{0}^{t}dt_{1}\sqrt{E(t_{1})}=\frac{1}{\sqrt{2}}\int_{0}^{t}dt_{1}\sqrt{\partial_{t_{1}}\left[tr(Q\partial_{t_{1}}\Sigma )\right]+2{\langle x^{\prime }\left(t_{1}\right)\rangle }^{T}Q\langle x^{\prime }\left(t_{1}\right)\rangle +\mathrm{tr}\left(Q^{\prime \prime }\Sigma \right)},$$

*where $Q=\Sigma^{-1}$ (recall, a prime denotes $\frac{\partial }{\partial t}$).*

**Proof.**  To prove this theorem, we use the PDF (5) in (4). To simplify the expression, we let

$$w\equiv \delta x=x-\langle x\left(t\right)\rangle ,\;\;Q=\Sigma^{-1}.$$

We then compute step by step $\frac{{\left[\partial_{t_{1}}p\left(x,t_{1}\right)\right]}^{2}}{p(x,t_{1})}$ as follows:

(12) $$\begin{array}{ccc} \;\;\;\;\;\partial_{t_{1}}p\left(x,t_{1}\right)\;\;\;\;\; & = & \;\;\;\;\;\frac{\partial }{\partial t_{1}}\left[{\left(\det(2\pi \Sigma )\right)}^{-\frac{1}{2}}e^{-\frac{1}{2}w^{T}Qw}\right]\\ \;\;\;\;\;\;\;\;\; & = & -\frac{1}{2}e^{-\frac{1}{2}w^{T}Qw}\;\;{\left(\det(2\pi \Sigma )\right)}^{-\frac{3}{2}}\partial_{t_{1}}\;\;\left(\det(2\pi \Sigma )\right)\;\;-\;\;\frac{1}{2}{\left(\det(2\pi \Sigma )\right)}^{-\frac{1}{2}}e^{-\frac{1}{2}w^{T}Qw}\partial_{t_{1}}\;\;\left(w^{T}Qw\right), \end{array}$$

(13) $$\begin{array}{ccc} {\left[\partial_{t_{1}}p\left(x,t_{1}\right)\right]}^{2}\;\;\;\;\;\; & = & \;\;\;\;\;\;\frac{1}{4}e^{-w^{T}Qw}{\left(\det\left(2\pi \Sigma \right)\right)}^{-3}{\left[\partial_{t_{1}}\det\left(2\pi \Sigma \right)\right]}^{2}+\frac{1}{4}{\left(\det(2\pi \Sigma )\right)}^{-1}e^{-w^{T}Qw}{\left(\partial_{t_{1}}\left[w^{T}Qw\right]\right)}^{2}\\ \;\;\;\;\;\; & & \;\;\;\;\;\;+\frac{1}{2}{\left(\det(2\pi \Sigma )\right)}^{-2}\partial_{t_{1}}\left[\det(2\pi \Sigma )\right]\partial_{t_{1}}\left[w^{T}Qw\right]e^{-w^{T}Qw}, \end{array}$$

(14) $$\begin{array}{ccc} \frac{{\left[\partial_{t_{1}}p\left(x,t_{1}\right)\right]}^{2}}{p(x,t_{1})}\;\;\;\;\;\; & = & \;\;\;\;\;\;\frac{1}{4}{\left(\det(2\pi \Sigma )\right)}^{-\frac{5}{2}}{\left[\partial_{t_{1}}\det\left(2\pi \Sigma \right)\right]}^{2}e^{-\frac{1}{2}w^{T}Qw}+\frac{1}{4}{\left(\det(2\pi \Sigma )\right)}^{-\frac{1}{2}}e^{-\frac{1}{2}w^{T}Qw}{\left(\partial_{t_{1}}\left(w^{T}Qw\right)\right)}^{2}\\ \;\;\;\;\;\; & & \;\;\;\;\;\;+\frac{1}{2}{\left(\det(2\pi \Sigma )\right)}^{-\frac{3}{2}}\partial_{t_{1}}\left(\det(2\pi \Sigma )\right)\partial_{t_{1}}\left(w^{T}Qw\right)e^{-\frac{1}{2}w^{T}Qw}. \end{array}$$

Now, using Equation (12) in Equation (14), we compute the integral $E\left(t_{1}\right)=\int_{-\infty }^{\infty }x\left(\frac{{\left[\partial_{t_{1}}p\left(x,t_{1}\right)\right]}^{2}}{p(x,t_{1})}\right)$ as follows

(15) $$\begin{array}{ccc} E(t_{1})\;\;\;\;\;\; & = & \;\;\;\;\;\;\int_{-\infty }^{\infty }p\left(x,t_{1}\right)\left(\frac{{\left(\det(2\pi \Sigma )\right)}^{-2}}{4}{\left[\partial_{t_{1}}\left(\det(2\pi \Sigma )\right)\right]}^{2}+\frac{{\left(\partial_{t_{1}}\left(w^{T}Qw\right)\right)}^{2}}{4}+\frac{\partial_{t_{1}}\left[\det(2\pi \Sigma )\right]\partial_{t_{1}}\left[w^{T}Qw\right]}{2\det(2\pi \Sigma )}\right)dx\\ \;\;\;\;\;\; & = & \;\;\;\;\;\;\langle {\left(\frac{\partial_{t_{1}}\left[\det(2\pi \Sigma )\right]}{2\det(2\pi \Sigma )}\right)}^{2}\rangle +\langle {\left(\frac{\partial_{t_{1}}\left[w^{T}Qw\right]}{2}\right)}^{2}\rangle +\langle \frac{\partial_{t_{1}}\left[\det(2\pi \Sigma )\right]\partial_{t_{1}}\left[w^{T}Qw\right]}{2\det(2\pi \Sigma )}\rangle . \end{array}$$

To calculate the three averages in (15), we use the properties $\int_{-\infty }^{\infty }e^{-\frac{1}{2}w^{T}Qw}w=\sqrt{\det(2\pi \Sigma )}$ [32], $\partial_{t_{1}}\left[e^{-\frac{1}{2}w^{T}Qw}\right]=-\frac{1}{2}e^{-\frac{1}{2}w^{T}Qw}\partial_{t_{1}}\left[w^{T}Qw\right]$ and $\partial_{t_{1}t_{1}}\left[e^{-\frac{1}{2}w^{T}Qw}\right]=-\frac{1}{2}\partial_{t_{1}t_{1}}\left[w^{T}Qw\right]e^{-\frac{1}{2}w^{T}Qw}+\frac{1}{4}e^{-\frac{1}{2}w^{T}Qw}{\left(\partial_{t_{1}}\left[w^{T}Qw\right]\right)}^{2}$. We then have

(16) $$\begin{array}{ccc} E(t_{1})\;\;\;\;\;\; & = & \;\;\;\;\;\;{\left(\frac{\partial_{t_{1}}\left[\det(2\pi \Sigma )\right]}{2\det(2\pi \Sigma )}\right)}^{2}+\int_{-\infty }^{\infty }p\left(x,t_{1}\right){\left(\frac{\partial_{t_{1}}\left[w^{T}Qw\right]}{2}\right)}^{2}dx+\frac{\partial_{t_{1}}\left[\det(2\pi \Sigma )\right]}{2\det(2\pi \Sigma )}\int_{-\infty }^{\infty }p\left(x,t_{1}\right)\partial_{t_{1}}\left[w^{T}Qw\right]dx\\ \;\;\;\;\;\; & = & \;\;\;\;\;\;\frac{1}{4}{\left(\frac{\partial_{t_{1}}\left[\det(2\pi \Sigma )\right]}{\det(2\pi \Sigma )}\right)}^{2}+\frac{1}{4{\left(\det(2\pi \Sigma )\right)}^{\frac{1}{2}}}\int_{-\infty }^{\infty }\left[4\partial_{t_{1}t_{1}}e^{-\frac{1}{2}w^{T}Qw}+2\partial_{t_{1}t_{1}}\left[w^{T}Qw\right]e^{-\frac{1}{2}w^{T}Qw}\right]dx\\ \;\;\;\;\;\; & & \;\;\;\;\;\;-2\frac{\partial_{t_{1}}\left[\det(2\pi \Sigma )\right]}{2{\left(\det(2\pi \Sigma )\right)}^{\frac{3}{2}}}\int_{-\infty }^{\infty }\partial_{t_{1}}\left[e^{-\frac{1}{2}w^{T}Qw}\right]dx\\ \;\;\;\;\;\; & = & \;\;\;\;\;\;\frac{1}{4}{\left(\frac{\partial_{t_{1}}\left[\det(2\pi \Sigma )\right]}{\det(2\pi \Sigma )}\right)}^{2}+\frac{\partial_{t_{1}t_{1}}\left[\sqrt{\det(2\pi \Sigma )}\right]}{\sqrt{\det(2\pi \Sigma )}}+\frac{1}{2\sqrt{\det(2\pi \Sigma )}}\int_{-\infty }^{\infty }\partial_{t_{1}t_{1}}\left[w^{T}Qw\right]e^{-\frac{1}{2}w^{T}Qw}dx\\ \;\;\;\;\;\; & & \;\;\;\;\;\;-\frac{\partial_{t_{1}}\left[\det(2\pi \Sigma )\right]}{{\left(\det(2\pi \Sigma )\right)}^{\frac{3}{2}}}\partial_{t_{1}}\sqrt{\det(2\pi \Sigma )}. \end{array}$$

Here

(17) $$\partial_{t_{1}t_{1}}\left[w^{T}Qw\right]=\sum_{i,j=1}^{n}\left[\partial_{t_{1}t_{1}}\left(q_{ij}w_{i}w_{j}\right)\right]=\sum_{i,j=1}^{n}\left[4q_{ij}^{\prime }w_{i}^{\prime }w_{j}+\underset{\mathrm{independent}\;\mathrm{of}\;x}{\underset{︸}{2q_{ij}w_{i}^{\prime }w_{j}^{\prime }}}+2q_{ij}w_{i}^{\prime \prime }w_{j}+\underset{w^{T}Q^{\prime \prime }w}{\underset{︸}{q_{ij}^{\prime \prime }w_{i}w_{j}}}\right].$$

We recall that $\omega_{i}^{\prime },q_{ij}^{\prime }$ and $\omega_{i}^{\prime \prime },q_{ij}^{\prime \prime }$ denote the first and second derivative over time of the elements $\omega_{i}$ and $q_{ij}$. By substituting (17) in (16) and making some arrangements, we obtain

(18) E(t1)=14∂t1det(2πΣ)det(2πΣ)2+∂t1t1det(2πΣ)det(2πΣ)+124∑i,j=1nqij′wi′wj0+12∑i,j=1n2qijwi″wj0+12∑i,j=1n2qijwi′wj′+12wTQ″w−12∂t1det(2πΣ)det(2πΣ)2.

Now with the help of the following relations $\langle w^{T}Q^{\prime \prime }w\rangle =\mathrm{tr}\left(Q^{\prime \prime }\Sigma \right)$ [33], $\partial_{t_{1}}\det\left(\Sigma \right)=\det\left(\Sigma \right)\mathrm{tr}\left(Q\partial_{t_{1}}\Sigma \right)$ [34], and

$\partial_{t_{1}t_{1}}\sqrt{\det(2\pi \Sigma )}=\frac{1}{4}\sqrt{\det(2\pi \Sigma )}{\left(\mathrm{tr}(Q\partial_{t_{1}}\Sigma )\right)}^{2}+\frac{1}{2}\sqrt{\det(2\pi \Sigma )}\partial_{t_{1}}\left(\mathrm{tr}\left(Q\partial_{t_{1}}\Sigma \right)\right)$, we then have

(19) $$\begin{array}{ccc} E(t_{1})\;\;\;\;\;\; & = & \;\;\;\;\;\;-\frac{1}{4}{\left(\mathrm{tr}(Q\partial_{t_{1}}\Sigma )\right)}^{2}+\frac{1}{2}\partial_{t_{1}}\left[\mathrm{tr}(Q\partial_{t_{1}}\Sigma )\right]+\frac{1}{4}{\left(\mathrm{tr}(Q\partial_{t_{1}}\Sigma )\right)}^{2}\\ \;\;\;\;\;\; & & \;\;\;\;\;\;+{\langle x^{\prime }\left(t_{1}\right)\rangle }^{T}Q\langle x^{\prime }\left(t_{1}\right)\rangle +\frac{1}{2}\mathrm{tr}\left(Q^{\prime \prime }\Sigma \right)\\ & = & \;\;\;\;\;\;\frac{1}{2}\partial_{t_{1}}\left[\mathrm{tr}(Q\partial_{t_{1}}\Sigma )\right]+{\langle x^{\prime }\left(t_{1}\right)\rangle }^{T}Q\langle x^{\prime }\left(t_{1}\right)\rangle +\frac{1}{2}\mathrm{tr}\left(Q^{\prime \prime }\Sigma \right). \end{array}$$

Equation (19) thus proves Equation (11). ☐

Given important properties of the covariance matrix eigenvalues (see, e.g., [35]), it is useful to express Equation (19) and the information length as a function of these covariance matrix eigenvalues. This is done in the following Corollary.

**Corollary** **1.** *Let $\varphi_{i}\left(t\right)$’s (i=1,...n) be the eigenvalues of the covariance matrix* Σ, *and $\bar{x}={\langle x^{\prime }\left(t\right)\rangle }^{T}P$ where P is an orthonormal matrix whose column vectors are linearly independent eigenvectors of $Q=\Sigma^{-1}$. We can rewrite the information length (11) as*

(20) $$L\left(t\right)=\int_{0}^{t}dt_{1}\sqrt{E(t_{1})}=\int_{0}^{t}dt_{1}\sqrt{\sum_{i=1}^{n}\left(\frac{{\varphi^{\prime }}_{i}^{2}\left(t_{1}\right)+2\varphi_{i}\left(t_{1}\right){\bar{x}}_{i}^{2}}{2\varphi_{i}^{2}\left(t_{1}\right)}\right)}.$$

**Proof.**  The proof follows straightforwardly from the fact that Σ is a symmetric matrix which can be diagonalised by finding the orthonormal matrix P such that $P^{T}\Sigma^{-1}P=\Phi$. Here Φ is the diagonal matrix whose entries are the eigenvalues $\frac{1}{\varphi_{i}\left(t\right)}$∀i=1,2,⋯,n (recall that $\varphi_{i}\left(t\right)$ is *i*-th the eigenvalue of $\Sigma^{-1}$). This gives us

(21) $$E\left(t\right)=\frac{1}{2}\sum_{i=1}^{n}\left(\partial_{t}\left[\frac{\varphi_{i}^{\prime }\left(t\right)}{\varphi_{i}\left(t\right)}\right]+\varphi_{i}\left(t\right)\partial_{tt}\left[\frac{1}{\varphi (t)}\right]+2\frac{{\bar{x}}_{i}^{2}}{\varphi_{i}\left(t\right)}\right)=\sum_{i=1}^{n}\left(\frac{{\varphi^{\prime }}_{i}^{2}\left(t\right)+2\varphi_{i}\left(t\right){\bar{x}}_{i}^{2}}{2\varphi_{i}^{2}\left(t\right)}\right).$$

This finishes the proof. ☐

It is useful to check that Equation (20) reproduces the previous result for the O-U process [36]

(22) $$E=\frac{\beta^{\prime 2}}{2\beta^{2}}+2\beta {\langle x^{\prime }\rangle }^{2},$$

where $\beta =\frac{1}{2\langle {\left(x-\langle x\rangle \right)}^{2}\rangle }$ is the inverse temperature. Here, $\beta^{\prime }$ denotes the time derivative of β. To show this, we note that for the O-U process, the covariance matrix is a scalar (n=1) with the value $\Sigma =\frac{1}{2\beta }=\varphi \left(t\right)$ and thus $Q=\frac{1}{\varphi (t)}=2\beta$ while $\langle x^{\prime }\left(t\right)\rangle =\langle x^{\prime }\rangle$. Thus,

$$E\left(t\right)=\frac{1}{2}{\left(\frac{-\frac{1}{2}\beta^{-2}\beta^{\prime }}{\frac{1}{2\beta }}\right)}^{2}+2\beta {\langle x^{\prime }\rangle }^{2}=\frac{\beta^{\prime 2}}{2\beta^{2}}+2\beta {\langle x^{\prime }\rangle }^{2}.$$

In sum, for the O-U process, the square of the information velocity (shown in expression (22)) increases with the ‘roughness’ of the process, as quantified by the squared ratio of the rate of change of the inverse temperature (or precision) and the precision – plus a term that depends upon this precision times the variance of the state velocity.

## 4. Kramers Equation

In this section we apply our results in Section 3 to the Kramers equation for a harmonically bound particle [19,37]. As noted in Introduction, we investigate the behaviour of the information length when varying various parameters and initial conditions to elucidate how the information geometry is affected by the damping, oscillations, strength of the stochastic noises and initial mean values.

Consider the Kramers equation

(23) $$\begin{array}{ccc} \frac{dx}{dt}\;\;\;\;\; & = & \;\;\;\;\;v\\ \frac{dv}{dt}\;\;\;\;\; & = & \;\;\;\;\;-\gamma v-\omega^{2}x+\xi \left(t\right). \end{array}$$

Here, ω is a natural frequency and γ is the damping constant, both positive real numbers. ξ is a Gaussian white-noise acting on *v* with the zero mean value 〈ξ(t)〉=0, with the statistical property

(24) $$\langle \xi \left(t\right)\xi \left(t_{1}\right)\rangle =2D\delta \left(t-t_{1}\right).$$

Comparing Equations (23) and (24) with Equations (2) and (3), we note that $x_{1}=x$, $x_{2}=v$, $\xi_{1}=0$, $\xi_{2}=\xi$, $D_{11}=0$, $D_{12}=0$, and $D_{22}=D$ while the matrix A for (23) has the element $A_{11}=0,A_{12}=1,A_{21}=-\omega^{2},A_{22}=-\gamma$. Thus, the eigenvalues of A are $\lambda_{1,2}=-\frac{1}{2}\left(\gamma \pm \sqrt{\gamma^{2}-4\omega^{2}}\right)$.

To find the information length for the system (23), we use Proposition 1 and Theorem 1. First, Proposition 1 requires the computation of the exponential matrix $e^{At}$ involving a rather long algebra with the help of [38]. The result is:

(25) $$\begin{array}{c} e^{At}\;\;=\;\;L^{-1}\;\;\left[{\left(sI-A\right)}^{-1}\right]\;\;=\;\;L^{-1}\;\;\left[\left[\begin{array}{cc} \frac{s+\gamma }{\left(s-\lambda_{1}\right)\left(s-\lambda_{2}\right)} & \frac{1}{\left(s-\lambda_{1}\right)\left(s-\lambda_{2}\right)}\\ -\frac{\omega^{2}}{\left(s-\lambda_{1}\right)\left(s-\lambda_{2}\right)} & \frac{s}{\left(s-\lambda_{1}\right)\left(s-\lambda_{2}\right)} \end{array}\right]\right]\;\;=\;\;\left[\begin{array}{cc} \frac{e^{\lambda_{1}t}\left(\gamma +\lambda_{1}\right)-e^{\lambda_{2}t}\left(\gamma +\lambda_{2}\right)}{\lambda_{1}-\lambda_{2}} & \frac{e^{\lambda_{1}t}-e^{\lambda_{2}t}}{\lambda_{1}-\lambda_{2}}\\ -\frac{\left(e^{\lambda_{1}t}-e^{\lambda_{2}t}\right)\omega^{2}}{\lambda_{1}-\lambda_{2}} & \frac{e^{\lambda_{1}t}\lambda_{1}-e^{\lambda_{2}t}\lambda_{2}}{\lambda_{1}-\lambda_{2}} \end{array}\right]. \end{array}$$

Here, $I\in R^{n\times n}$ is the identity matrix. Similarly, we can show

(26) $$\begin{array}{c} 2\int_{0}^{t}e^{A(t-t_{1})}De^{A^{T}\left(t-t_{1}\right)}dt_{1}=\\ \left[\begin{array}{cc} \frac{D\left(\frac{-1+e^{2\lambda_{1}t}}{\lambda_{1}}+\frac{-\lambda_{1}-4e^{(\lambda_{1}+\lambda_{2})t}\lambda_{2}+3\lambda_{2}+e^{2\lambda_{2}t}\left(\lambda_{1}+\lambda_{2}\right)}{\lambda_{2}\left(\lambda_{1}+\lambda_{2}\right)}\right)}{{\left(\lambda_{1}-\lambda_{2}\right)}^{2}} & \frac{D{\left(e^{\lambda_{1}t}-e^{\lambda_{2}t}\right)}^{2}}{{\left(\lambda_{1}-\lambda_{2}\right)}^{2}}\\ \frac{D{\left(e^{\lambda_{1}t}-e^{\lambda_{2}t}\right)}^{2}}{{\left(\lambda_{1}-\lambda_{2}\right)}^{2}} & \frac{D\left(\left(-1+e^{2\lambda_{2}t}\right)\lambda_{2}+\lambda_{1}\left(-\frac{4e^{(\lambda_{1}+\lambda_{2})t}\lambda_{2}}{\lambda_{1}+\lambda_{2}}+\frac{4\lambda_{2}}{\lambda_{1}+\lambda_{2}}+e^{2\lambda_{1}t}-1\right)\right)}{{\left(\lambda_{1}-\lambda_{2}\right)}^{2}} \end{array}\right]. \end{array}$$

Using Equations (25) and (26) in Equations (6) and (7), we have the time-dependent (joint) PDF (5) at any time *t* for our system (23) and (24). To calculate Equation (11) with the help of Equations (25) and (26), we perform numerical simulations (integrations) for various parameters in Equations (23) and (24) as well as initial conditions. Note that while we have simulated many different cases, for illustration, we show some representative cases by varying *D*, ω, γ and 〈x(0)〉, 〈v(0)〉 in Section 4.1, Section 4.2 and Section 4.3 and Appendix A, respectively, for the same initial covariance matrix Σ(0) with elements $\Sigma_{11}\left(0\right)=\Sigma_{22}\left(0\right)=0.01$ and $\Sigma_{12}\left(0\right)=\Sigma_{21}\left(0\right)=0$. Note that the initial marginal distributions of p(x(0)) and p(v(0)) are Gaussian with the same variance 0.01. Results in the limit ω→0 are presented in Section 4.4.

### 4.1. Varying *D*

**Figure 1:**
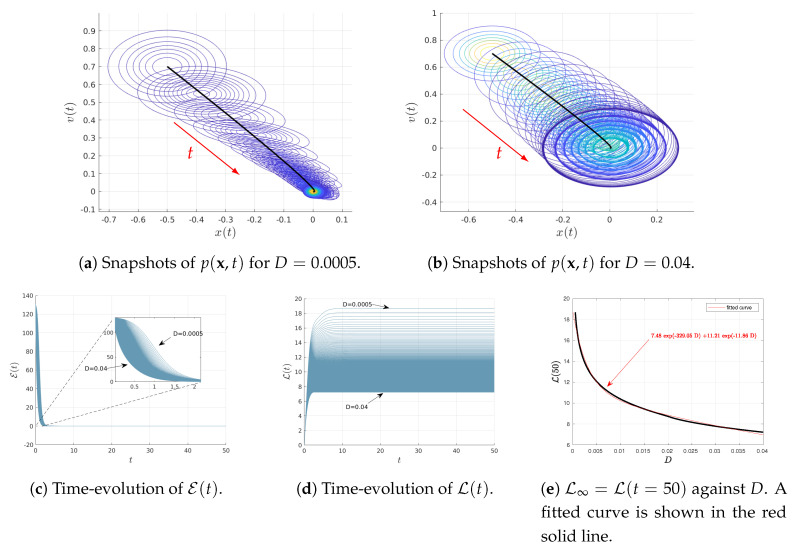
Results of Equations (23) and (24) for 〈x(0)〉=−0.5, 〈v(0)〉=0.7, γ=2, ω=1, D∈(0.0005,0.04) and the initial covariance matrix Σ(0) with elements $\Sigma_{11}(0)=\Sigma_{22}(0)=0.01,\;\Sigma_{12}(0)=\Sigma_{21}(0)=0$ .

Figure 1 shows the results when varying *D* as D∈(0.0005,0.04) for the fixed parameters γ=2 and ω=1. The initial joint PDFs are Gaussian with the fixed mean values 〈x(0)〉=−0.5, 〈v(0)〉=0.7; as noted above, the covariance matrix Σ(0) with elements $\Sigma_{11}\left(0\right)=\Sigma_{22}\left(0\right)=0.01$ and $\Sigma_{12}\left(0\right)=\Sigma_{21}\left(0\right)=0$. Consequently, at t=0, the marginal distributions of p(x(0)) and p(v(0)) are Gaussian PDFs with the same variance 0.01 and the mean values 〈x(0)〉=−0.5 and 〈v(0)〉=0.7, respectively.

Figure 1a,b show the snapshots of time-dependent joint PDF p(x,t) (in contour plots) for the two different values of D=0.0005 and D=0.04, respectively. The black solid represents the phase portrait of the mean value of 〈x(t)〉 and 〈v(t)〉 while the red arrows display the direction of time increase. Note that in Figure 1b, only some of the initial snapshots of the PDFs are shown for clarity, given the great amount of overlapping between different PDFs. Figure 1c,d show the time-evolution of the information velocity E(t) and information length L(t), respectively, for different values of D∈(0.0005,0.04). It can be seen that the system approaches a stationary (equilibrium) state for t≳20 for all values of *D*, L(t) approaching constant values (recall L(t) does not change in a stationary state). Therefore, we approximate the total information length as $L_{\infty }=L\left(t=50\right)$, for instance. Finally, the total information length $L_{\infty }=L\left(t=50\right)$ is shown in Figure 1e. We determine the dependence of $L_{\infty }$ on *D* by fitting an exponential function as $L_{\infty }\left(D\right)=7.84e^{-329.05D}+11.21e^{-11.86D}$ (shown in red solid line).

### 4.2. Varying ω or γ

**Figure 2:**
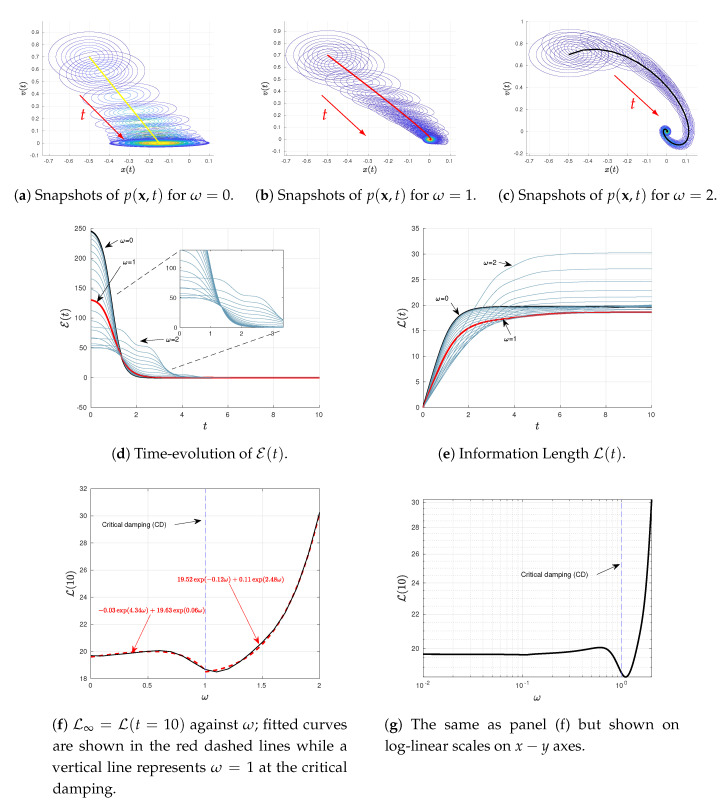
Results of Equations (23) and (24) for 〈x(0)〉=−0.5, 〈v(0)〉=0.7, γ=2, ω∈(0,2), D∈0.0005, and the initial covariance matrix Σ(0) with elements $\Sigma_{11}(0)=\Sigma_{22}(0)=0.01,\;\Sigma_{12}(0)=\Sigma_{21}(0)=0$.

**Figure 3:**
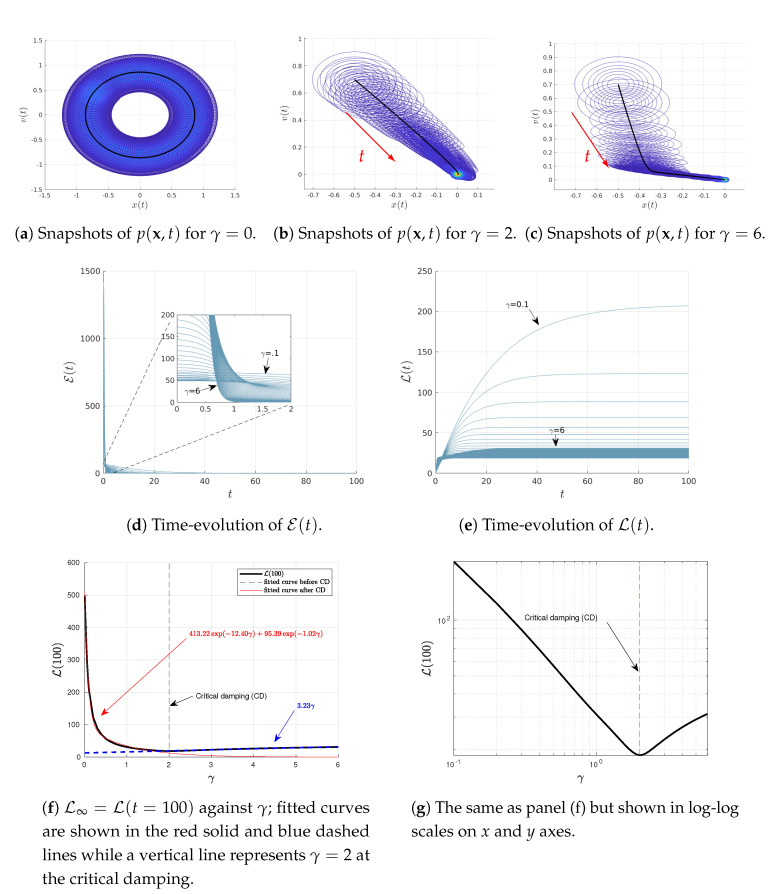
Results of Equations (23) and (24) for 〈x(0)〉=−0.5, 〈v(0)〉=0.7, γ∈(0,6), ω=1, D=0.0005, and the initial covariance matrix Σ(0) with elements $\Sigma_{11}(0)=\Sigma_{22}(0)=0.01,\Sigma_{12}(0)=\Sigma_{21}(0)=0$.

We now explore how results depend on the two parameters ω and γ, associated with oscillation and damping, respectively. To this end, we use D=0.0005 and the same initial conditions as in Figure 1 but vary ω∈(0,2) and γ∈(0,6) in Figure 2 and Figure 3, respectively. Specifically, in different panels of these figures, we show the snapshots of the joint PDF p(x,t), the time-evolutions of E(t) and L(t) for different values of ω∈(0,2) and γ∈(0,6), and $L_{\infty }$ against either ω or γ. From Figure 2e and Figure 3e, we can see that the system is in a stationary state for sufficiently large t=10 and t=100, respectively. Thus, we use $L_{\infty }=L\left(t=10\right)=L\left(10\right)$ in Figure 2f,g and $L_{\infty }=L\left(t=100\right)=L\left(10\right)$ in Figure 3f,g.

Notably, Figure 2f,g (shown on linear-linear and log-linear scales on x−y axes, respectively) exhibit an interesting a non-monotonic dependence of $L_{\infty }$ on ω for the fixed γ=2, with the presence of a distinct minimum in $L_{\infty }$ at certain ω. Similarly, Figure 3f,g (shown in linear-linear and log-log scales on x−y axes, respectively) also shows a non-monotonic dependence of $L_{\infty }$ on γ for the fixed ω=1. These non-monotonic dependences are more clearly seen in Figure 2g and Figure 3g. A close inspection of these figures then reveals that the minimum value of $L_{\infty }$ occurs close to the critical damping (CD) γ∼2ω; specifically, this happens at ω∼1 for γ=2 in Figure 2f,g while at γ∼2 for ω=1 in Figure 3f,g. We thus fit $L_{\infty }$ against ω or γ depending on whether ω or γ is smaller/larger than its critical value as follows:

(27) $$\begin{array}{ccc} L_{10}\left(\omega \right) & = & -0.03e^{4.34\omega }+19.63e^{0.06\omega }\;\forall \;\omega \in \left(0,1\right), \end{array}$$

(28) $$\begin{array}{ccc} L_{10}\left(\omega \right) & = & 19.52e^{-0.12\omega }+0.11e^{2.48\omega }\;\forall \;\omega \in \left(1,2\right), \end{array}$$

(29) $$\begin{array}{ccc} L_{100}\left(\gamma \right) & = & 413.22e^{-12.4\gamma }+95.39e^{-1.02\gamma }\;\forall \;\gamma \in \left(0,2\right), \end{array}$$

(30) $$\begin{array}{ccc} L_{100}\left(\gamma \right) & = & 3.23\gamma \;\forall \;\gamma \in (2,6). \end{array}$$

The fitted curves in Equations (27)–(30) are superimposed in Figure 2f and Figure 3f, respectively. It is important to notice from Equations (27)–(30) that $L_{\infty }$ tends to increase as either ω→∞ for a finite, fixed γ (<∞) or γ→∞ for a finite, fixed ω (<∞).

Finally, we note that for the critical damping γ=2ω, the eigenvalue becomes a real double root with the value $\lambda_{1,2}\to -\omega$. Thus, in this limit, we have that

(31) $$\langle x\left(t\right)\rangle =\left[\begin{array}{c} e^{-t\omega }\left(x\left(0\right)+t\left(v\left(0\right)+\left(\gamma -\omega \right)x\left(0\right)\right)\right)\\ e^{-t\omega }\left(-tx\left(0\right)\omega^{2}-tv\left(0\right)\omega +v\left(0\right)\right) \end{array}\right],$$

and Σ(t) is composed by the following elements

(32) $$\begin{array}{ccc} \Sigma_{11}\left(t\right) & = & \frac{e^{-2t\omega }\left(2\omega^{3}\left(\Sigma_{11}{\left(\gamma t-t\omega +1\right)}^{2}+t^{2}\left(\left(\Sigma_{12}+\Sigma_{21}\right)\left(\gamma -\omega \right)+\Sigma_{22}\right)+t\left(\Sigma_{12}+\Sigma_{21}\right)\right)+D\left(-2t\omega \left(t\omega +1\right)+e^{2t\omega }-1\right)\right)}{2\omega^{3}},\\ \Sigma_{12}\left(t\right) & = & e^{-2t\omega }\left(t\left(-\omega^{2}\left(\Sigma_{11}\gamma t+\Sigma_{11}+\Sigma_{21}t\right)+\Sigma_{11}t\omega^{3}-\Sigma_{22}t\omega +\Sigma_{22}+Dt\right)-\Sigma_{12}\left(t\omega -1\right)\left(\gamma t-t\omega +1\right)\right),\\ \Sigma_{21}\left(t\right) & = & e^{-2t\omega }\left(t\left(-\omega^{2}\left(\Sigma_{11}\gamma t+\Sigma_{11}+\Sigma_{12}t\right)+\Sigma_{11}t\omega^{3}-\Sigma_{22}t\omega +\Sigma_{22}+Dt\right)-\Sigma_{21}\left(t\omega -1\right)\left(\gamma t-t\omega +1\right)\right),\\ \Sigma_{22}\left(t\right) & = & \frac{e^{-2t\omega }\left(2t\omega^{2}\left(t\omega^{2}\left(\Sigma_{11}\omega +\Sigma_{12}+\Sigma_{21}\right)-\omega \left(\Sigma_{12}+\Sigma_{21}\right)+\Sigma_{22}\left(t\omega -2\right)\right)+2\Sigma_{22}\omega +D\left(-2t\omega \left(t\omega -1\right)+e^{2t\omega }-1\right)\right)}{2\omega }. \end{array}$$

Equations (31) and (32) are used in Section 4.1 (Figure 1).

### 4.3. Varying 〈x(0)〉 or 〈v(0)〉

**Figure 4:**
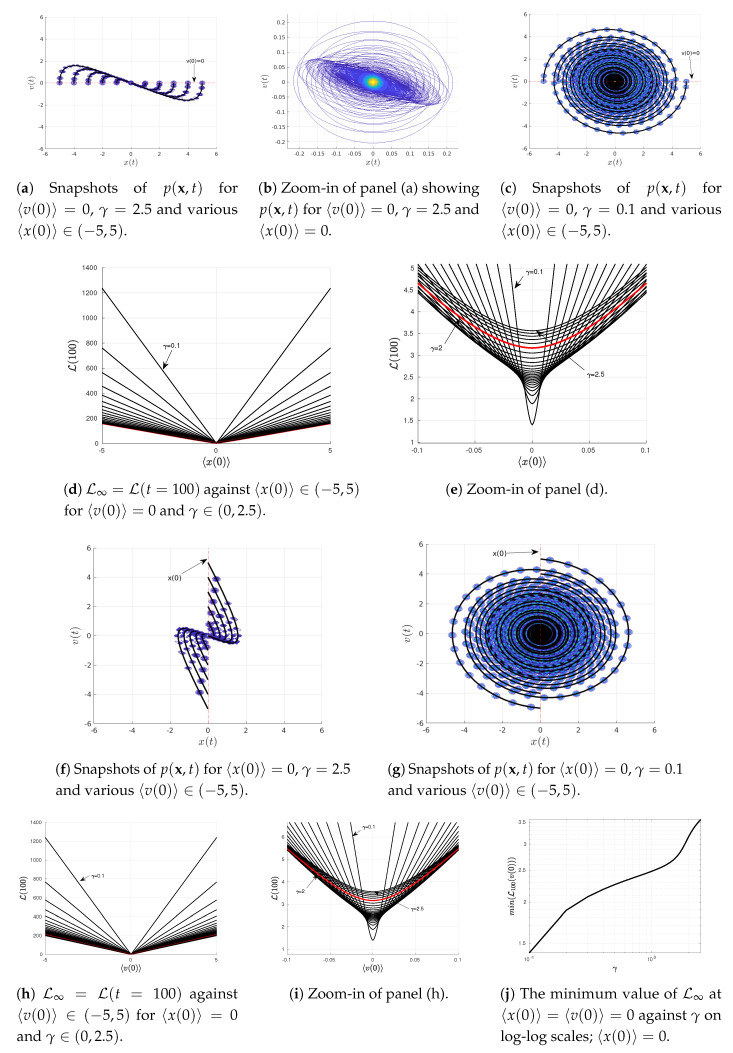
Results of Equations (23) and (24) scanned over 〈x(0)〉∈(−5,5) for 〈v(0)〉=0 [Figure 4a–e] and 〈v(0)〉∈(−5,5) for 〈x(0)〉∈=0 [Figure 4f–j]. The parameter values ω=1,D=0.0005, and γ∈(0,2.5) while the initial covariance matrix Σ(0) has the elements $\Sigma_{11}(0)=\Sigma_{22}(0)=0.01,\Sigma_{12}(0)=\Sigma_{21}(0)=0$.

To elucidate the information geometry associated with the Kramer equation (Equations (23) and (24)), we now investigate how $L_{\infty }$ behaves near the equilibrium point 〈x(0)〉=〈v(0)〉=0. To this end, we scan over 〈x(0)〉 for 〈v(0)〉=0 in Figure 4a–e while scanning over 〈v(0)〉 for 〈x0)〉=0 in Figure 4f–i. For our illustrations in Figure 4, we use the same initial covariance matrix Σ(0) as in Figure 1, Figure 2 and Figure 3, D=0.0005 and ω=1 and a few different values of γ (above/below/at the critical value γ=2). We note that the information geometry near a non-equilibrium point is studied in Appendix A.

Specifically, snapshots of p(x,t) are shown in Figure 4a–f for γ=2.5 (above its critical value γ=2=2ω) while those in Figure 4c–g are for γ=0.1 below the critical value 2. By approximating $L_{\infty }=L\left(t=100\right)$, we then show how $L_{\infty }$ depends on 〈x(0)〉 and 〈v(0)〉 for different values of γ in Figure 4d,e and Figure 4h,i, respectively.

Figure 4d,e show the presence of a minimum in $L_{\infty }$ at the equilibrium 〈x(0)〉=0 (recall 〈v(0)〉=0); $L_{\infty }$ is a linear function of 〈x(0)〉 for 〈x(0)〉≫0.1, which can be described as $L_{\infty }\left(x\left(0\right),\gamma \right)=h\left(\gamma \right)\left|\langle x\left(0\right)\rangle \right|+f\left(\gamma \right)$. Here, h(γ) and f(γ) are constant functions depending on γ for a fixed ω which represent the slope and the *y*-axis intercept, respectively. A non-zero value of $L_{\infty }$ at 〈x(0)〉=0 is caused by the adjustment (oscillation and damping) of the width of the PDFs in time due to the disparity between the width of the initial and equilibrium PDFs (see Figure 4b). In other words, even though the mean values remain in equilibrium for all time ${\left[\langle x\left(0\right)\rangle ,\langle v\left(0\right)\rangle \right]}^{T}=\lim_{t\to \infty }\langle x\left(t\right)\rangle ={\left[0,0\right]}^{T}$, the information length (11) depends on the covariance matrix Σ which changes from its initial value to the final equilibrium value as follows

$$\Sigma \left(0\right)=\left[\begin{array}{cc} 0.01 & 0\\ 0 & 0.01 \end{array}\right]\;\mathrm{to}\;\lim_{t\to \infty }\Sigma \left(t\right)=\left[\begin{array}{cc} \frac{D}{\gamma \omega^{2}} & 0\\ 0 & \frac{D}{\gamma } \end{array}\right].$$

On the other hand, $L_{\infty }$ against 〈x(0)〉 shows parabolic behaviour for small 〈x(0)〉<0.1 in Figure 4e. This is caused by the finite width $0.1=\sqrt{\Sigma_{11}\left(0\right)}=\sqrt{\Sigma_{22}\left(0\right)}$ of the initial p(x,0); we see that 〈x(0)〉<0.1 is within the uncertainty of the initial p(x,0).

Similarly, Figure 4h,i exhibit a minimum in $L_{\infty }$ at the equilibrium 〈v(0)〉=0 (recall 〈x(0)〉=0 in this case); $L_{\infty }$ is a linear function of 〈v(0)〉 for 〈v(0)〉≫0.1 described by $L_{\infty }\left(v\left(0\right),\gamma \right)=H\left(\gamma \right)|\langle v\left(0\right)|+F\left(\gamma \right)$ (again parabolic for 〈v(0)〉<0.1, see Figure 4i). Here again, H(γ) and F(γ) are constant functions depending on γ for a fixed ω which represent the slope and the *y*-axis intercept, respectively.

Finally, Figure 4j shows in logarithmic scale that the minimum value of $L_{\infty }$ at 〈x(0)〉=〈v(0)〉 monotonically increases with γ.

### 4.4. The Limit Where ω→0

When the natural frequency ω=0 (i.e., damped-driven system like the O-U process [36]) in Equation (23), the two eigenvalues of the matrix A become $\lambda_{1}\to -\gamma$ and $\lambda_{2}\to 0$. It then easily follows that

(33) $$\langle x\left(t\right)\rangle =\left[\begin{array}{c} \frac{v\left(0\right)-e^{-\gamma t}v\left(0\right)}{\gamma }+x\left(0\right)\\ e^{-\gamma t}v\left(0\right) \end{array}\right],$$

and Σ(t) is composed by the elements

(34) $$\begin{array}{ccc} \Sigma_{11}\left(t\right) & = & \frac{e^{-2\gamma t}\left(-D+\Sigma_{22}\left(0\right)\gamma +e^{\gamma t}\left(4D-\gamma \left(2\Sigma_{22}\left(0\right)+\left(\Sigma_{12}\left(0\right)+\Sigma_{21}\left(0\right)\right)\gamma \right)\right)+e^{2\gamma t}\left(\gamma \left(\Sigma_{22}\left(0\right)+\gamma \left(\Sigma_{12}\left(0\right)+\Sigma_{21}\left(0\right)+\Sigma_{11}\left(0\right)\gamma \right)\right)+D\left(2\gamma t-3\right)\right)\right)}{\gamma^{3}},\\ \Sigma_{12}\left(t\right) & = & \frac{e^{-2\gamma t}\left(D{\left(-1+e^{\gamma t}\right)}^{2}-\Sigma_{22}\left(0\right)\gamma +e^{\gamma t}\gamma \left(\Sigma_{22}\left(0\right)+\Sigma_{12}\left(0\right)\gamma \right)\right)}{\gamma^{2}},\\ \Sigma_{21}\left(t\right) & = & \frac{e^{-2\gamma t}\left(D{\left(-1+e^{\gamma t}\right)}^{2}-\Sigma_{22}\left(0\right)\gamma +e^{\gamma t}\gamma \left(\Sigma_{22}\left(0\right)+\Sigma_{21}\left(0\right)\gamma \right)\right)}{\gamma^{2}},\\ \Sigma_{22}\left(t\right) & = & \frac{e^{-2\gamma t}\left(D\left(-1+e^{2\gamma t}\right)+\Sigma_{22}\left(0\right)\gamma \right)}{\gamma }. \end{array}$$

**Figure 5:**
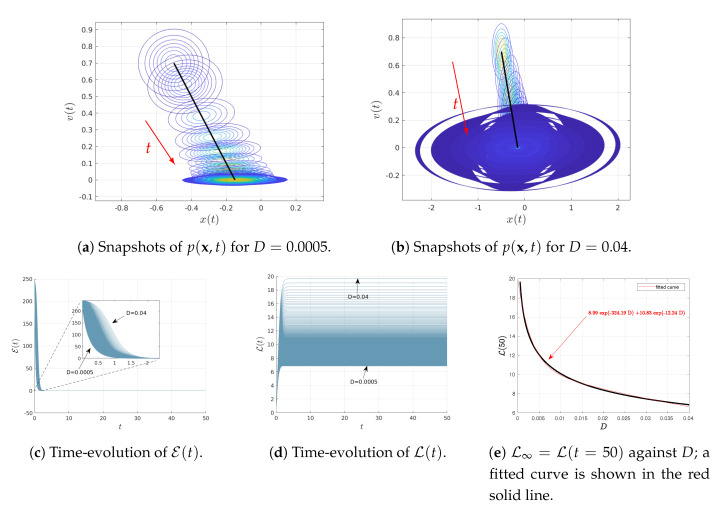
Results of Equations (23) and (24) for 〈x(0)〉=−0.5, 〈v(0)〉=0.7, γ=2, ω=0, D∈(0.0005,0.04) and the initial covariance matrix Σ(0) with elements $\Sigma_{11}(0)=\;\Sigma_{22}(0)=0.01,\;\Sigma_{12}(0)=\Sigma_{21}(0)=0$.

To investigate the case of ω→0, we consider the scan over D∈(0.0005,0.04) for the same parameter value γ=2, and the initial conditions as in Figure 1, apart from using ω=0 instead of ω=1. Figure 5 presents the results – snapshots of p(x,t), time evolutions of E(t), L(t), and $L_{\infty }=L\left(t=50\right)$ against *D* in Figure 5a–e. In particular, in Figure 5e, we identify the dependence of $L_{\infty }$ on *D* by fitting the results to the curve $L_{=}8.99e^{-324.19D}+10.83e^{-12.24D}$.

## 5. Concluding Remarks

We have presented theoretical results of time-dependent PDFs and the information length for *n*-th order linear autonomous stochastic processes, which can be applied to a variety of practical problems. In particular, the information length diagnostics was found as a function of the mean and covariance matrices; the latter was further expressed in terms of the covariance matrix eigenvalues. A Specific application was made to a harmonically bound particle system with the natural oscillation frequency ω, subject to a damping γ and a Gaussian white-noise (Kramer equation). We investigated how the information length depends on ω and γ, elucidating the role of critical damping γ=2ω in information geometry. The fact that the information length tends to take its minimum value near the critical damping can be viewed as the simplification of dynamics and thus the decrease in information change due to the reduction of the two characteristic time scales associated with ω and γ to the one value. On the other hand, the information length in the long time limit was shown to preserve the linear geometry associated with the Gaussian statistics in a linear stochastic process, as in the case of the O-U process.

Future works would include the exploration of our results when applied to high-dimensional processes and the extension of our work to a more general (e.g., finite-correlated) stochastic noise, non-autonomous systems or non-linearly coupled systems. In particular, it will be of interest to look for a geodesic solution in non-autonomous systems [9] with the help of an external force, optimization or guiding self-organization (multi-agent systems) as well as elucidating the role of critical damping and resonances in self-organization. In addition, it would also be interesting to utilize the results introduced in [39] to predict the bound on the evolution of any observable for the Kramers problem (23), and compare it with a natural observable in such a system, the energy, for instance.

## Figures and Tables

**Figure A1:**
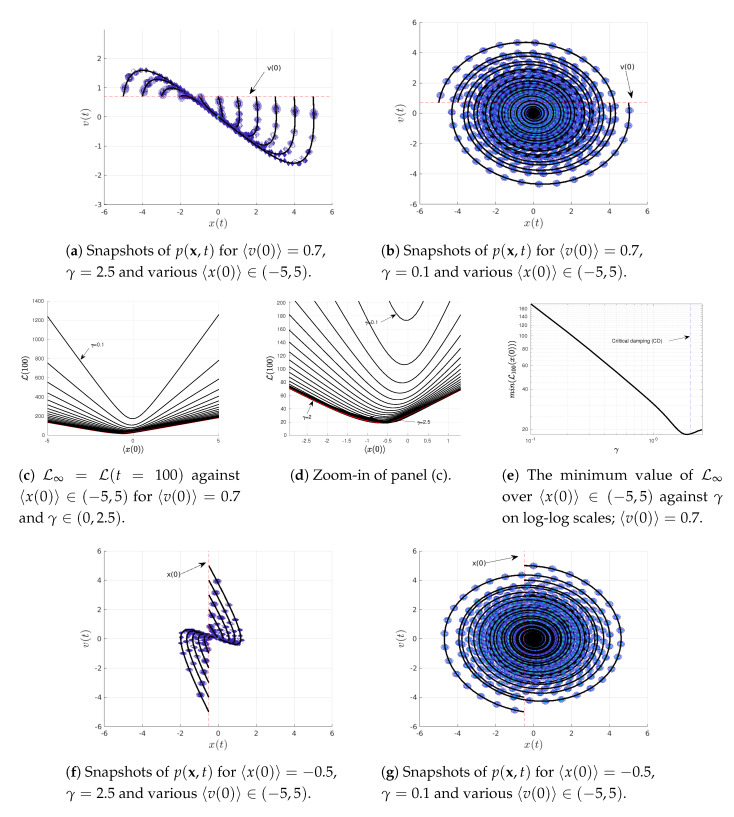

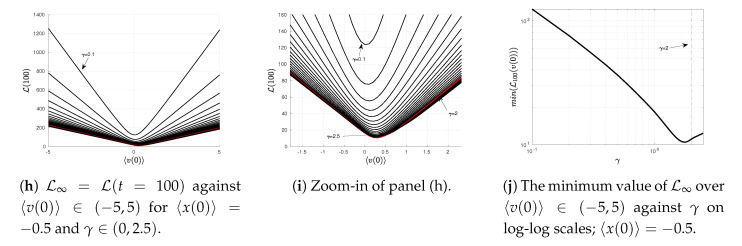
Results of Equations (23) and (24) scanned over 〈x(0)〉∈(−5,5) for 〈v(0)〉=0.7 [Figure A1a–e] and 〈v(0)〉∈(−5,5) for 〈x(0)〉=−0.5 [Figure A1f–j]. The parameter values ω=1, D=0.0005, and γ∈(0,2.5) while the initial covariance matrix Σ(0) has the elements $\Sigma_{11}\left(0\right)=\Sigma_{22}\left(0\right)=0.01$, $\Sigma_{12}\left(0\right)=\Sigma_{21}\left(0\right)=0$.

## Appendix A. Analysis for Non-Zero Fixed Initial Conditions

In Section 4.3 we analysed the behaviour of the information geometry associated with the Kramer equation (Equations (23) and (24)) for different γ∈(0,2.5) near the equilibrium point 〈x(0)〉=〈v(0)〉=0. To this end, we plotted $L_{\infty }$ when varying 〈x(0)〉 and 〈v(0)〉 for a fixed 〈v(0)〉=0 and 〈x(0)〉=0, respectively. In this Appendix, we want to show how such information geometry changes near a non-equilibrium point by scanning over 〈x(0)〉 and 〈v(0)〉 for a fixed non-zero 〈v(0)〉=0.7 and 〈x(0)〉=−0.5, respectively. We show that the use of non-zero fixed initial conditions changes the location of the minimum $L_{\infty }$ depending on γ. Here, we use the same parameter values D=0.0005, ω=1, $\Sigma_{12}\left(0\right)=\Sigma_{21}\left(0\right)=0$ and $\Sigma_{11}\left(0\right)=\Sigma_{22}\left(0\right)=0.01$.

First, snapshots of p(x,t) are shown in Figure A1a,f for γ=2.5 (above its critical value γ=2=2ω) while those in Figure A1b,g are for γ=0.1 below the critical value 2. It is important to notice that there is a non-symmetric behaviour of the trajectories of the system for γ≫0. This is shown at Figure A1a,f whose trajectories asymmetrically vary over the initial conditions in comparison with the results shown in Figure 4a,f. By approximating $L_{\infty }=L\left(t=100\right)$, we then show how $L_{\infty }$ depends on 〈x(0)〉 and 〈v(0)〉 for different values of γ in Figure A1c,d and Figure A1h,i, respectively. Of prominence in Figure A1c,d is the presence of a distinct minimum in $L_{\infty }$ for a particular value of $\langle x\left(0\right)\rangle =x_{c}$, $L_{\infty }$ linearly increasing with $|\langle x\left(0\right)\rangle -x_{c}|$ for a sufficiently large $|\langle x\left(0\right)\rangle -x_{c}|$; similarly, Figure A1h,i shows a distinct minimum in $L_{\infty }$ for a particular value of $\langle v\left(0\right)\rangle =v_{c}$, $L_{\infty }$ linearly increasing with $|\langle v\left(0\right)\rangle -v_{c}|$ for a sufficiently large $|\langle v\left(0\right)\rangle -v_{c}|$.

Finally, we scan over 〈x(0)〉 and 〈v(0)〉 and identify the minimum value of $L_{\infty }$ for a given γ and plot this minimum value of $L_{\infty }$ (at $x_{c}$ and $v_{c}$) against γ in Figure A1e,j. In Figure A1e,j, $L_{\infty }$ against γ takes its minimum near the critical damping γ=2ω=2 (shown in a vertical line), as observed previously in Section 4.1 and Section 4.2. This is clearly different from the behaviour of the minimum value of $L_{\infty }$ against γ (for the equilibrium point 〈x(0)〉=0 and 〈v(0)〉=0) in Figure 4j where $L_{\infty }$ monotonically increases with γ. This is because for 〈x(0)〉=0 and 〈v(0)〉=0, mean values does not change over time, with less effect of oscillations (ω) and thus the critical damping γ=2ω.
